# Evaluation of the level of serum Interleukins (IL-2, IL-4, IL-15 andIL-17) and its relationship with disease severity in patients with alopecia areata^☆^^☆☆^

**DOI:** 10.1016/j.abd.2021.03.006

**Published:** 2021-07-17

**Authors:** Özge Aşkın, Sera Nur Yücesoy, Erkam Coşkun, Burhan Engin, Server Serdaroğlu

**Affiliations:** Istanbul University Cerrahpasa Medical Faculty, Istanbul, Turkey

**Keywords:** Alopecia, Alopecia areata, Interleukins

## Abstract

**Background:**

Alopecia areata (AA) is a hair disease that causes hair loss without scarring. The etiopathogenesis of AA has not been fully understood yet.

**Objective:**

To determine serum interleukin levels (IL-2, IL-4, IL-15, and IL-17) in patients diagnosed with alopecia areata and to investigate the relationship of IL levels with the duration and severity of alopecia areata and the response to tofacitinib therapy.

**Methods:**

Patients (≥16 years old) diagnosed with alopecia areata and healthy individuals as a control group was enrolled. Baseline serum interleukin levels of the patients and controls were measured. In the patient group receiving tofacitinib therapy, serum interleukin levels were measured again after 6 months. Disease severity for alopecia areata was assessed using the Severity of Alopecia Tool.

**Results:**

Sixty-one AA patients and 30 healthy individuals were included; they were comparable regarding age and sex. The mean disease duration for AA was 7 ± 6 years and the baseline mean Severity of Alopecia Tool score was 71 ± 30 (range, 20–100). Baseline IL-2, IL-4 and IL-15 levels were significantly higher in the patient group than those in the control group (p < 0.001 for each). No significant correlation was found between the baseline interleukin levels and either disease duration or disease severity (baseline Severity of Alopecia Tool score). Among the patients receiving tofacitinib (n = 22), all interleukin levels significantly decreased after treatment. However, no significant relationship between the change in interleukin levels and the change in the Severity of Alopecia Tool scores was observed after tofacitinib treatment.

**Study limitations:**

This is a monocentric study conducted in a single university hospital.

**Conclusion:**

High interleukin levels in alopecia areata patients and the significant decrease with treatment support the idea that interleukins have a role in pathogenesis. Nevertheless, no relationship could be demonstrated between IL levels and disease duration or severity.

## Introduction

Alopecia Areata (AA) is a disease that causes hair loss without scarring. The disease targets the epithelium of hair follicles and involves hair-bearing areas of the head and the other parts of the body.^1^ The prevalence of AA in the general population is estimated to be 0.1%–0.2% and the life-long risk of developing AA is estimated to be nearly 1.7%.^1^ The clinical presentation of AA can be as one or multiple patches of hair loss on the scalp or, much rarer, as complete baldness (alopecia totalis) or total loss of body hair (alopecia universalis).^2^ Disease severity is assessed by using the Severity of Alopecia Tool (SALT) scores.^3^

The etiopathogenesis of AA has not been fully clarified yet. In their study, Petukhova et al. showed that genetic background has a role in the pathogenesis.^4^ In addition to genetic background, environmental and immune factors are considered to have a role.^5^, ^6^ There is growing evidence that it is a polygenic autoimmune disease. Earlier studies have demonstrated a relationship between AA and HLA alleles, some DQB and DR alleles, and Interleukin (IL)-1 cluster genes.^6^

In the treatment of severe AA, there is a limited response to traditional approaches, and the search for effective treatment is ongoing. Treatments that target different immune system pathways are being introduced as new therapeutic options.^7^ Case reports, case series, and open-label studies on the use of Tofacitinib, a Janus kinase (JAK) 1 and 3 inhibitors, have been published in recent years.^8^ Previous studies showed that interleukins especially IL-2, IL-4, and IL-15 may have a role in the pathogenesis of AA. Current studies also mention IL-17 as a potential pro-inflammatory cytokine having a role in the disease mechanism. Tofacitinib is JAK inhibitor that interferes with JAK1 and JAK3. JAK3 interacts with the cytokine receptor, the cyc, which is specifically used for some interleukin receptors including IL-2, IL-4, IL15.^9^ Also, tofacitinib inhibits Th17 cells that produce IL-17.^10^

The present study aimed to determine the serum levels of interleukins (IL-2, IL-4, IL-15, and IL-17), which are likely to play a role in the pathogenesis in patients diagnosed with AA. In comparison to previous studies, the relationship of interleukin levels with the duration and severity (SALT score) of AA and the response to tofacitinib therapy was also investigated.

## Methods

Patients aged ≥16 years old, who had been clinically diagnosed with AA between 01.09.2019 and 01.06.2020 at the study’s faculty were enrolled in the study. Some patients had hair loss in their bodies in addition to hair loss in the scalp. Patients with acute or chronic critical illnesses that may hinder the study, who are pregnant or in lactation period, receiving any drugs that may interfere with interleukin levels, and those who had received any treatment including topical therapy for AA in the last 2 months were excluded. Acute or chronic critical illnesses include any organ dysfunction or presence of an infection that needs to be treated or followed. The control group consisted of healthy individuals without any acute or chronic disease. They were willing to take part in the research and recruited from public domains. Approval of the Ethics Committee (IUC Date and nº 07/08/2019-121094) and informed consents of all participants were obtained before the study.

Demographic and clinical information of the patients were inquired. Baseline serum IL levels of the patients and the controls were measured. In the patient group receiving tofacitinib therapy, serum IL levels were measured again after 6 months of treatment. Patients with AA who were resistant to topical therapy were started to tofacitinib therapy after taking their informed consents. Before starting the treatment with tofacitinib, hemogram, liver function tests, kidney function tests, amylase, lipase levels, viral serology, quantiferon test, and chest-X-Ray were asked from all patients. Blood tests were repeated every month and patients were followed in the study’s clinic. The daily dose of tofacitinib was 10 mg/day.

Disease severity for AA was assessed using the SALT. In the SALT scoring system, the scalp is divided into 4 areas; vertex corresponds to 40%, right profile corresponds to 18%, left profile corresponds to 18% and occipital region corresponds to 24% of the scalp surface area. After determining the percentage of hair loss in these 4 regions, it is multiplied by the percentage value that corresponds to the relevant area, and then the SALT score is obtained as the sum of the scores obtained from each of the 4 areas. Higher scores indicate severe disease; total hair loss on the scalp corresponds to 100 points.^3^

Peripheral blood samples taken from the patients and the controls to analyze serum interleukin levels were centrifuged at 3000 rpm for 10 minutes, and then the serum was separated and stored at −30 °C until the time of analysis. Serum IL-2, IL-4, IL-15, and IL-17 levels were measured by the ELISA method. The values were presented as ng/L.

### Statistical analysis

Data were analyzed using the PASW Statistics for Windows, Version 18.0 (SPSS Inc., Chicago, IL, USA). The suitability of variables to normal distribution was analyzed by visual (histogram and probability graphics) and analytic methods (Kolmogorov-Smirnov/Shapiro-Wilk tests). Descriptive statistics were presented as numbers and percentages for categorical variables and as median, percentile 25 (Q1) and percentile 75 (Q3) for numerical variables. For categorical variables, paired group comparison was performed using the Chi-Square test. As normal distribution condition was not provided, the Mann-Whitney *U* test was used in paired group comparison for numerical variables. Pre-post analyses for each group were performed using the Wilcoxon Signed Rank test. The relationship between numerical variables was analyzed by Spearman’s rho test. The situations where type 1 error was below 5% were interpreted as statistically significant.

## Results

The study included 61 AA patients and 30 healthy volunteers as the control group. The patient group and the control group were comparable in terms of age and sex (Table 1). The mean disease duration for AA was 7 ± 6 years and the baseline mean SALT score was 71 ± 30 (range, 20–100). Tofacitinib treatment was used in 52.5% of the patients. In the remaining group, 36.1% of patients were started other treatments including topical immunotherapy and systemic steroid treatment.

**Table 1:** General characteristics of alopecia areata patients and the controls.

	Patient group (n = 61)	Control group (n = 30)	p
**Age, years**	30 ± 10	30 ± 7	0.493
	28 (20–38)	29.5 (25–34)	
**Sex**			
Male	30 (49.2)	15 (50.0)	0.941
Female	31 (50.8)	15 (50.0)	
**Disease duration, years**	7 ± 6 5 (3–10)	–	–
**SALT score**	71 ± 30 80 (40–100)	–	–
**Treatment**			
Tofacitinib	32 (52.5)	–	–
SADBE	17 (27.9)	–	–
Steroid	4 (6.6)	–	–
DPCP	1 (1.6)	–	–
None	7 (11.5)	–	–

The values are presented as number (%) or mean ± standard deviation, and median (Q1–Q3).

DPCP, Diphenylcyclopropenone; SADBE, Squaric Acid Dibutylester; SALT, Severity of Alopecia Tool.

Serum IL concentrations of the patients and the control group are demonstrated in Table 2. Baseline IL-2, IL-4, and IL-15 levels were significantly higher in the patient group as compared to those of the control group.

**Table 2:** Baseline interleukin levels of the patient and control groups.

Interleukins, ng/L	Patient group (n = 61)	Control group (n = 30)	p
	Mean ± SD	Mean ± SD	
	Median (Q1–Q3)	Median (Q1–Q3)	
IL-2	709 ± 747	308 ± 150	< 0.001
	359.53 (306.34–709.42)	285.63 (216.03–344.66)	
IL-4	544 ± 439	183 ± 110	< 0.001
	324.99 (273.87–648.9)	150.08 (99.1716–251.26)	
IL-15	485 ± 636	168 ± 184	< 0.001
	195.1 (136.39–496.33)	134.66 (65.46–170.49)	
IL-17	158 ± 189	109 ± 69	0.723
	80.66 (60.27–162.38)	83.56 (56.69–171.12)	

IL, Interleukin; SD, Standard Deviation.

IL levels were analyzed according to the genders in the patient group and the control group. No significant difference was determined between males and females in terms of IL levels both in the patient and control groups (Table 3).

**Table 3:** Interleukin levels according to the sex.

	n	Male	n	Female	p
		Mean ± SD		Mean ± SD	
		Median (Q1–Q3)		Median (Q1–Q3)	
**Control Group**					
IL-2	15	297 ± 137	15	319 ± 166	0.724
		292.72 (189–361.58)		284 (224.42–327.89)	
IL-4	15	201 ± 133	15	166 ± 82	0.663
		149.30 (99.17–254.26)		150.86 (87.64–251.26)	
IL-15	15	225 ± 244	15	110 ± 60	0.178
		151.39 (80.31–294.1)		118.69 (48–144.1)	
IL-17	15	101 ± 52	15	117 ± 83	0.820
		73.54 (61.04–170.59)		99.81 (45.08–202.42)	
**Patient Group**					
IL-2	30	742 ± 662	31	678 ± 831	0.260
		378.24 (314.86–911.03)		348.47 (290.95–462.66)	
IL-4	30	605 ± 463	31	486 ± 413	0.337
		344.15 (276.71–1146.33)		303.53 (262.56–536.01)	
IL-15	30	504 ± 631	31	467 ± 651	0.937
		193.78 (130.28–590.95)		200.42 (139.63–446.04)	
IL-17	30	148 ± 152	31	168 ± 222	0.920
		85.03 (57.33–192.49)		78.97 (62.49–124.5)	

IL, Interleukin; SD, Standard Deviation.

No statistically significant correlation was found between baseline IL levels and either disease duration or disease severity (baseline SALT score) (Table 4).

**Table 4:** Correlation between baseline interleukin levels and disease duration and disease severity (Severity of Alopecia Tool score).

Baseline IL levels	Baseline SALT score			Disease duration		
	n	rho	p	n	rho	p
IL-2	61	0.070	0.593	61	0.072	0.579
IL-4	61	-0.117	0.370	61	0.104	0.427
IL-15	61	-0.109	0.404	61	0.059	0.650
IL-17	61	-0.073	0.576	61	0.149	0.253

IL, Interleukin; SALT, Severity of Alopecia Tool.

There were 32 patients receiving tofacitinib. Pre- and post-treatment IL levels and SALT scores were compared in 22 of these patients, in whom the measurements were repeated at the 6^th^ month. A significant decrease was found in all IL levels after treatment when compared to those before treatment (baseline). Additionally, a significant decrease was observed in disease severity assessed by SALT score (Table 5).

**Table 5:** Interleukin levels and Severity of Alopecia Tool scores in the patients receiving tofacitinib treatment.

	Pre-treatment (n = 22)	Post-treatment (n = 22)	p
	Median (Q1–Q3)	Median (Q1–Q3)	
**SALT score**	100 (50–100)	40 (24–50)	< 0.001
**IL level**			
IL-2	346.92 (314.08–462.66)	248.96 (174.55–298.89)	< 0.001
IL-4	317.16 (276.71–504.72)	152.0 (128.08–290.31)	0.022
IL-15	208.86 (125.64–490.64)	122.82 (68.36–200.4)	0.033
IL-17	88.69 (64.88–107.74)	49.32 (38.77–82.34)	0.016

IL, Interleukin; SALT, Severity of Alopecia Tool.

There was no significant relationship between the change in IL levels and the change in the SALT scores after treatment in comparison to that before treatment in the patients receiving tofacitinib (Table 6).

**Table 6:** Relationship between the change in interleukin levels and the change in Severity of Alopecia Tool scores in the patients receiving tofacitinib treatment.

	Difference in SALT Scores (Post–Pre)		
	n	rho	p
**Difference in IL-2 (Post-Pre)**	22	0.199	0.376
**Difference in IL-4 (Post–Pre)**	22	-0.296	0.180
**Difference in IL-15 (Post**–**Pre)**	22	-0.169	0.452
**Difference in IL-17 (Post–Pre)**	22	-0.137	0.545

IL, Interleukin; SALT, Severity of Alopecia Tool.

## Discussion

Depending on disease severity, the psychosocial status of AA patients is influenced, and their quality of life is impaired.^11^ The absence of a curative treatment for this disease leads to the continuation of the search for efficient treatment and studies to illuminate the pathogenesis. It is thought that immunological mechanisms are effective in the pathogenesis of AA and that various cytokines such as Interferon (IFN), Tumor Necrotizing Factor (TNF), and interleukins as well play a role.^12^, ^13^

There is a limited number of clinical studies investigating the relationship of interleukins with disease severity and its role in the pathogenesis of AA. In the present study, the authors evaluated the levels of IL-2, IL-4, IL-15, and IL-17. Baseline IL-2, IL-4, and IL-15 levels were significantly higher in the patient group than those in the control group (p < 0.001 for each). No significant correlation was found between the baseline interleukin levels and either disease duration or disease severity. There was no statistically significant difference between IL-17 levels of the patient and control group and no significant correlation between IL-17 levels and duration or severity of AA.

IL-2 is a cytokine playing a role in immune memory with a proven relationship with autoimmune conditions, and the use of IL-2 or its receptors in immunotherapy is debatable.^14^ Kasumagić-Halilovic et al.^15^ found higher IL-2 levels in AA patients as compared to that of the controls. No correlation was found between IL-2 levels and clinical type of AA or disease duration. Likewise, the authors also found higher IL-2 levels in AA patients than that in the controls. The authors found no significant correlation between IL-2 levels and duration or severity of AA.

IL-4 is an interleukin that takes part in various physiological and pathological processes and plays a role in tissue adhesion and inflammation.^16^ Kalkan et al.^17^ concluded that IL-4 VNTR polymorphism might be a risk factor for the development of AA. In the present study, IL-4 levels were higher in AA patients when compared to that of the controls (p < 0.001). There was no significant correlation between IL-4 levels and duration or severity of AA.

IL-15 is a multifunctional cytokine with a documented relationship with autoimmune diseases.^18^ Ebrahim et al.^19^ reported higher serum IL-15 levels in AA patients as compared to the controls and a significantly positive correlation between SALT scores and IL-15 levels. They concluded that IL-15 might be a marker of AA severity. In the present study, likewise, IL-15 levels were higher in AA patients in comparison to that of the controls (p < 0.001). However, no significant correlation was found between IL-15 levels and duration or severity of AA.

IL-17 is a cytokine that plays a critical role in the epithelial barrier of the body parts that are frequently exposed to microorganisms, as well as in immunopathology, host defense, and inflammation.^20^ El-Morsy et al.^21^ determined higher serum IL-17A levels in AA patients as compared to the controls; nevertheless, they reported the absence of a significant correlation between IL-17A levels and gender, disease duration, SALT score or disease severity. Based on these findings, they concluded that IL-17A potentially plays a role in the pathogenesis but does not affect disease severity. In another study, no difference was found between IL-17A levels of AA patients and of the controls; however, patients’ IL-17A levels showed a significant decrease after Narrowband-Ultraviolet B treatment.^22^ Loh et al.^23^ reported higher serum IL-17 levels in AA patients as compared to the controls in their study. In their review, Ramot et al.^24^ concluded that there is no clear relationship between IL-17 levels and disease severity or duration despite higher IL-17 levels reported in AA patients. In the present study, although IL-17 level was higher in AA patients than the controls, the difference was not statistically significant. Furthermore, there was no significant correlation between IL-17 levels and duration or severity of AA.

Drug selection for the treatment of AA depends on numerous factors such as patient’s age, extensiveness/duration of the disease, patient’s expectation, treatment cost, and presence of comorbidity. Although there is no curative treatment yet, various therapies are being implemented to provide remission. Corticosteroids, topical minoxidil, topical immunotherapy (DPCP, SADBE), anthralin, phototherapy, immunosuppressive agents, prostaglandin analogs, biologics, and JAK inhibitors are among the therapies that are currently being implemented and considered beneficial.^25^ Recently, along with increasing information on the pathogenesis and the introduction of the role of the JAK pathway, the therapeutic potential of JAK inhibitors (tofacitinib, ruxolitinib and baricitinib) in AA began to attract attention.^26^ Beneficial effects of tofacitinib in the treatment of moderate-severe AA have been reported in case series and clinical studies.^27^, ^28^, ^29^, ^30^, ^31^ Guo et al.^32^ carried out a systematic review and a meta-analysis and reported that tofacitinib is beneficial in the treatment of AA patients and causes only mild adverse events, and they recommended verification of these data in future randomized, large-scale clinical trials. In the present study, interleukin levels were re-studied, and the SALT score was re-measured at the 6^th^ month of treatment in 22 of the AA patients receiving tofacitinib. While the patients’ median (Q1–Q3) SALT score was 100 (50–100) at baseline, it became 40 (24–50) at the 6^th^ month of tofacitinib therapy. This decrease in disease severity was found statistically significant (p < 0.001). The interleukin levels of the patients studied in the present study demonstrated significant reduction with tofacitinib therapy.

In conclusion, high interleukin levels in AA patients and the significant decrease with treatment support the idea that interleukins have a role in pathogenesis. Nevertheless, no relationship could be demonstrated between IL levels and disease duration or severity. Further studies with larger patient populations are required. Understanding the role of interleukins in pathogenesis will make a contribution to the development of new targeted therapies.

## Conclusion

High interleukin levels in alopecia areata patients and the significant decrease following tofacitinib treatment support the idea that interleukins have a role in the pathogenesis of AA. Further studies are needed to investigate the role of interleukins in the pathogenesis of AA.

## Financial support

None declared.

## Authors' contributions

Özge aşkın: Approval of the final version of the manuscript; critical literature review; data collection, analysis and interpretation; effective participation in research orientation; ıntellectual participation in propaedeutic and/or therapeutic management of studied cases; manuscript critical review; preparation and writing of the manuscript; statistical analysis; study conception and planning.

Sera Nur Yücesoy: Approval of the final version of the manuscript; critical literature review; data collection, analysis and interpretation; effective participation in research orientation; ıntellectual participation in propaedeutic and/or therapeutic management of studied cases; manuscript critical review; preparation and writing of the manuscript.

Erkam Coşkun: Approval of the final version of the manuscript; critical literature review; data collection, analysis and interpretation; effective participation in research orientation.

Burhan Engin: Approval of the final version of the manuscript; critical literature review; data collection, analysis and interpretation; effective participation in research orientation.

Server Serdaroğlu: Approval of the final version of the manuscript; critical literature review; data collection, analysis and interpretation; effective participation in research orientation; ıntellectual participation in propaedeutic and/or therapeutic management of studied cases; manuscript critical review; preparation and writing of the manuscript.

## Conflicts of interest

None declared.
